# Authors' reply to letters from Egilman *et al* and Oliver *et al*

**DOI:** 10.1136/oemed-2016-103870

**Published:** 2016-07-27

**Authors:** Julian Peto, Clare Gilham, Christine Rake, Andrew Darnton, John Hodgson, Garry Burdett

**Affiliations:** 1 London School of Hygiene and Tropical Medicine, London, UK; 2 Health and Safety Executive, Bootle, UK; 3 Health and Safety Laboratory, Buxton, UK

**Keywords:** Lung cancer, Dose-response

Dr Egilman and colleagues claim our report^1^ implies that chrysotile does not cause mesothelioma. So did the International Chrysotile Association, the website of which highlighted our paper with the misleading headline ‘reliable scientific data confirms negligible role of chrysotile in UK patients with asbestos-related lung disease’.^2^ Our results are consistent with the strong evidence that chrysotile did not cause a large proportion of UK mesotheliomas, but they certainly do not show that the risk is negligible. We said ‘the rapid clearance of chrysotile from the lung with a half-life of a few months explains its virtual absence in our samples, and implies that we cannot estimate its effects except by noting that amphibole lung burdens account very well for mesothelioma incidence’. In relation to lung cancer, we said, ‘the contribution of chrysotile to current UK lung cancer rates is not known and may be impossible to ascertain’. Uncontrolled chrysotile use caused large lung cancer and asbestosis risks, and its abandonment by Europe and many other countries stimulated development of novel alternatives rather than causing economic damage. These are sufficient grounds for worldwide replacement of chrysotile with safer substitutes. The mesothelioma risk, although less, reinforces the case.

Dr Egilman and colleagues also criticise our analysis of lung rather than pleural tissue and our restriction of transmission electron microscope (TEM) counting to asbestos fibres longer than 5 μm. We observed that ‘for fibres of specified dimension it seems reasonable to assume a linear relationship between inhaled dose, fibre concentration in pleural stomata and our measurements in lung parenchyma’. After reviewing the human, animal and in vitro evidence, an expert panel convened by the US Agency for Toxic Substances and Disease Registry concluded that ‘There is a strong weight of evidence that asbestos and synthetic vitreous fibers shorter than 5 μm are unlikely to cause cancer in humans’.^3^ Our protocol for anonymised sample preparation used minimal ultrasonic treatment and did not involve freezedrying, and we used a single laboratory. They cite the assumption in 1978 that mesotheliomas in the Rochdale factory were caused by chrysotile.^4^ This predated the admission by the company that over 10 000 tonnes of crocidolite were also processed between 1932 and 1968.^5^

Dr Oliver and colleagues missed several details. There were no next-of-kin or proxy interviews, our statistical definition of asbestos-related lung cancer did not involve asbestosis (which would be biased) and we showed that our estimated ratio of mesothelioma to asbestos-related lung cancer is consistent with two population-based UK studies. That ‘the gold standard for asbestos-exposure assessment is the occupational history, not fiber burden’ is contradicted by the dose–response we observed within occupational groups and is particularly unhelpful in relation to Britain's high mesothelioma rate in both sexes due to environmental asbestos exposure from unidentified sources.^6^ The strong correlation we observed between lung burden and mesothelioma risk will not ‘imperil the diagnosis of asbestos-related disease, victim compensation, and public health measures aimed at primary and secondary prevention’. Our results, together with lung burdens in younger people, will enable the risks associated with current exposures from asbestos in buildings to be estimated, informing effective disease prevention.

## References

[1] Gilham C, Rake C, Burdett G, et al Pleural mesothelioma and lung cancer risks in relation to occupational history and asbestos lung burden. Occup Environ Med 2016;73:290–9. 10.1136/oemed-2015-103074 26715106 PMC4853597

[2] http://www.chrysotileassociation.com/en/news/n_list.php (accessed 20 May 2016).

[3] Eastern Research Group. Report on the Expert Panel on Health Effects of Asbestos and Synthetic Vitreous Fibers: the influence of fiber length. Prepared for: Agency for Toxic Substances and Disease Registry. Massachusetts: Eastern Research Group, 2003. https://www.atsdr.cdc.gov/hac/asbestospanel/finalpart1.pdf (accessed 16 June 2016).

[4] Peto J The hygiene standard for chrysotile asbestos. Lancet 1978;1:484–9. 10.1016/S0140-6736(78)90145-9 76030

[5] Peto J, Doll R, Hermon C, et al Relationship of mortality to measures of environmental asbestos pollution in an asbestos textile factory. Ann Occup Hyg 1985;29:305–55. 10.1093/annhyg/29.3.305 4073702

[6] Rake C, Gilham C, Hatch J, et al Occupational, domestic and environmental mesothelioma risks in the British population: a case-control study. Br J Cancer 2009;100:1175–83. 10.1038/sj.bjc.6604879 19259084 PMC2669989

